# News Feed Advertising and Positive Attitude: An Interpretation Model Based on Information Processing

**DOI:** 10.3389/fpsyg.2021.724140

**Published:** 2021-11-11

**Authors:** Qinglong Du, Yanxia Yang, Ying Liu, Qiqi He

**Affiliations:** ^1^School of Economics and Management, Southwest University of Science and Technology, Mianyang, China; ^2^Chengdu Sport University, Chengdu, China; ^3^Fisher College of Business, The Ohio State University, Columbus, OH, United States

**Keywords:** news feed advertising, brand attitude, brand experience, self-brand connection, advertising attitude

## Abstract

This study mainly examines the different effects and mechanisms of news feed advertising (vs. traditional display advertising) *on advertising attitude and brand attitude by using an observational experiment that categorized participants based on their past experiences*. By analyzing the video advertising of the “Li Ning” brand, three conclusion were drawn. First, compared with traditional display advertising, news feed advertising led to more positive advertising attitude and brand attitude. Second, generation and transmission mechanism of attitudes were applicable to the interpretation rules of the classical information processing process model, which showed that the information processing process of the news feed advertising and the traditional display advertising were consistent. Third, consumer’s brand experience did not affect the direct effects of “attention” on “memory,” but affected the mediate role of “interpretation,” which showed that news feed advertising may have a stronger “drainage” effect when promoting new products and developing new markets. However, if the consumer’s experience is not enhanced, the brand attitude triggered by news feed advertising will still not be stable. These findings are conducive to further understanding the effect of news feed advertising on advertising attitude and brand attitude.

## Introduction

News feed advertising is native advertising that is embedded in the information of the information streaming media platform and mixed with the functions of the Internet products (Fulgoni and Lipsman, 2014). Among them, news feed advertising, embedded in social communication media platforms, news streaming platforms, and short video streaming platforms, (e.g., Tiktok) has achieved rapid growth (Aribarg and Schwartz, 2020). In addition to becoming a new engine of advertising revenue in the Mobile Internet Era (Grewal et al., 2016; Zeng et al., 2017), well-designed news feed advertising is more likely to strengthen the connection between the brands and consumers, enhance the brand image, and thus expand the market share. Compared with traditional display advertising, news feed advertising is more effective and less invasive, which better meets consumer’s preference for advertising under the trend of social information acquisition, decision-making scenarios and fragmented behavior in the Mobile Internet Era (Martins et al., 2019). However, despite the prevalence of news feed advertising, we know very little about its effectiveness (Aribarg and Schwartz, 2020). Is news feed advertising really more effective than traditional advertising in influencing brand attitudes? If it’s more effective, what is the underlying mechanism? These are the main problems to be discussed in this study.

This study draws upon the Persuasion Knowledge Model to develop our theoretical framework. Persuasion Knowledge Model believes that the easier it is to detect the motivation hidden in the information, the easier it is to arouse consumer’s persuasion knowledge, thus triggering consumer’s resistance mechanism (Marian and Peter, 1994). First, news feed advertising is consistent with the surrounding environment, and it can lead to a more positive brand attitude. Second, news feed advertising has higher information processing fluency, and it can cause stronger self-brand connection, then establish a positive and stable brand attitude. Third, brand experience defines the boundary conditions for the effect of advertising attitude, and individuals with high brand experience are more likely to generate positive brand attitude.

Therefore, drawing upon the literature on Persuasion Knowledge Model (Marian and Peter, 1994) and processing fluency theory (Lee and Labroo, 2004), this study examines the effects of news feed advertising on brand attitude that was mediated by advertising attitude and self-brand connection, and also tests the moderating effect of brand experience. By hypothesizing and testing these relationships, this study makes some important contributions to the literatures on advertising and brand, offering a more balanced perspective that recognizes both the strengths and weaknesses of news feed advertising. First, this study enriched Persuasion Knowledge Model (Marian and Peter, 1994) and processing fluency theory (Lee and Labroo, 2004) by proving the positive results of news feed advertising. Specifically, this study combined the two theories and discussed in detail how and why news feed advertising affect brand attitude. Second, this study further contributes to the literature of news feed advertising and brand attitude by being among the first researcher to examine this positive relationship. Third, this study improves the mediate mechanism and boundary condition of news feed advertising by demonstrating why and when these effects occur. Specifically, there is little knowledge regarding the psychological mechanisms and boundary condition that explain the impact of news feed advertising on brand attitude. In terms of why, this study hypothesizes that advertising attitude and self-brand connection are the mechanism underlying the positive association of news feed advertising with brand attitude. In terms of when, this study identifies the brand experience for which these effects will be the strongest or will dissipate.

## Literature Review and Hypothesis

### News Feed Advertising

It has become a trend for consumers to get information through mobile phones, which has led to a fundamental change in the way consumers interact with brands. The traditional brand centered vertical communication paradigm has little effect, while the consumer centered horizontal communication paradigm is popular (Wollschlaeger et al., 2017), and news feed advertising has flourished.

News feed advertising first appeared on Facebook in 2006 and is a new type of advertising in the era of social media. It is embedded in the information of major social media platforms and integrated with the platform. What’ more, it takes platform characteristics as the main feature, takes the form of platform information as the main method, and uses big data algorithms to target users (C. Campbell and Evans, 2018; Kanuri et al., 2018). In recent years, news feed advertising embedded in social communication media platforms (such as Wechat Moments, MicroBlog), news streaming media platforms (such as Toutiao) and short video streaming media platforms (such as Tiktok) have grown particularly and become an important tool for brand communication. News feed advertising differs from traditional display advertising in two main aspects: location and appearance (Aribarg and Schwartz, 2020). As for the perspective of location, traditional display advertising is at a disadvantage because it is at the edge of the site and is often in short supply (Drèze and Hussherr, 2003; Hervet et al., 2011). Instead, news feed advertising is placed within other editorial content. As for the perspective of appearance, news feed advertising is consistent with its surrounding environment, which some literatures call originality (Wojdynski and Evans, 2016; C. Campbell and Evans, 2018). If it’s not for the “sponsorship” or “advertisement” surrounding it, it is hard for users to identify it as an advertisement (Wojdynski and Evans, 2016). Therefore, compared with the traditional display advertising, news feed advertising has the advantages of giving valuable information to the users at the same time not to undermine the user experience, and it also can enhance the fluency of consumer information processing (Campbell and Evans, 2018), and ultimately lead to more likely to be clicked (Sahni and Nair, 2020).

### The Influence of News Feed Advertising on Brand Attitude

Persuasion Knowledge Model believes that the easier it is to detect the motivation hidden in the information, the easier it is to arouse consumer’s persuasion knowledge, thus triggering consumer’s resistance mechanism (Marian and Peter, 1994). Advertising can evoke consumer’s persuasive knowledge (Campbell and Kirmani, 2000; Kirmani and Zhu, 2007; Wei et al., 2008), trigger critical thinking (Sophie C Boerman et al., 2014), and reduce brand attitudes (Sophie C. Boerman et al., 2012, 2015; Campbell and Evans, 2018). However, contrary to traditional display advertising, news feed advertising is consistent with the surrounding environment, and it is hard for consumers to find that it is actually an advertising. In other words, news feed advertising hides its persuasive intention from consumers to some extent, which makes it difficult for consumers to arouse their persuasive knowledge and skeptical knowledge when dealing with news feed advertising, thus generating less resistance and less negative attitude toward the brand. As a result, we believe that news feed advertising evokes a more positive brand attitude than traditional display advertising. Therefore, the following hypothesis is proposed:

Hypothesis 1: News feed advertising is more likely to lead to a more positive brand attitude than traditional display advertising.

### The Mediating Role of Advertising Attitude

Processing fluency theory points out that people’s perception of how easy it is to process information affects their response to information. It is believed that information with high perceived fluency is more likely to cause positive feedback from consumers (Lee and Labroo, 2004). As mentioned above, because of the consistency of the news feed advertising with the surrounding environment, it is hard for users to find that it is actually an advertising, which can convey the brand message without destroying the user experience. Therefore, it can improve the fluency of consumer information processing and thus arouse consumer’s positive feedback of brand attitude. Similarly, Wojdynski and Evans (2016) believe that news feed advertising hides its persuasive intention from consumers to some extent through moderate content originality. It is difficult for consumers to arouse their persuasive knowledge and skeptical knowledge when dealing with news feed advertising. Thus, it is easier for consumers to generate positive feedback to news feed advertising. Moreover, Ferreira et al. (2017) pointed out that prominent advertising forms can make consumers perceive higher goal impediment when processing information, which will lead to avoidance of advertising. However, the advertising forms integrated with the background are more easily accepted by consumers, and consumers deal with them as their main tasks, resulting in better advertising cognition and brand memory. Therefore, the following hypothesis is proposed:

Hypothesis 2a: News feed advertising is more likely to lead to more positive advertising attitude than traditional display advertising.

Hypothesis 2b: Advertising attitude mediates the relationship between news feed advertising and consumer brand attitudes.

### The Mediating Role of Self-Brand Connection

Self-brand connection refers to the extent to which consumers absorb the brand into their self-concept. Strong self-brand connection may lead to robust brand attitudes, that is, attitudes that are not very susceptible to change (Escalas and Bettman, 2013). The research of Wang and John (2019) on product upgrading also showed that customers with strong self-brand connection are more likely to choose product upgrading rather than directly abandon the brand when faced with the invasion of unfamiliar groups.

Establishing a positive advertising attitude through appropriate advertising forms (e.g., narrative processing) allows consumers to have more positive associations with the brand (Escalas, 2004). In other words, a positive advertising attitude can lead to a stronger self-brand connection among consumers. Compared with traditional display advertising, news feed advertising has higher information processing fluency, less interference and more concealment of persuasion intention, and it can cause stronger self-brand connection. Once a strong self-brand connection is established, consumers will reject brands that are inconsistent with the ingroup, thus establishing a positive and stable brand attitude (Escalas and Bettman, 2005). Therefore, the following hypothesis is proposed:

Hypothesis 3: Self-brand connection mediates the relationship between advertising attitude and brand attitude.

### The Moderating Role of Brand Experience

Brand experience refers to the subjective internal reaction (feeling, emotion, and cognition) and behavioral reaction of consumers caused by brand-related stimuli (Brakus et al., 2009). These reactions are all subjective knowledge and consist of a multi-dimensional structure including consumer cognition, emotion, behavior, and reaction to enterprises and society. Good brand experience can lead to better brand attitude and brand loyalty (Brakus et al., 2009). Hoffman and Novak (2018) proposed that, it can promote consumer’s self-extension and self-expansion when experience is enabling experience. Consumers with brand experience activate brand-related codes stored in their brains when receiving advertising information, and the indirect meaning associated with advertising is activated and spreading activation, thereby producing more positive or negative attitudes and emotions (Sheth and Solomon, 2014). In addition, in order to avoid cognitive dissonance, under the same advertising attitude, consumers with brand experience (especially those who are using the brand) are more inclined to generate positive brand attitude and brand-self association compared with consumers without brand experience. Therefore, the following hypothesis is proposed:

Hypothesis 4a: Brand experience moderates the relationship between advertising attitude and brand attitude, such that the positive relationship will be stronger when brand experience is high rather than low.

Hypothesis 4b: Brand experience moderates the mediating effect of self-brand connection between advertising attitude and brand attitude, such that the indirect effect will be stronger when brand experience is high rather than low.

**FIGURE 1:**
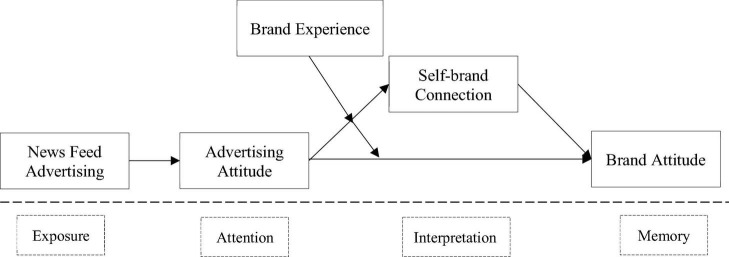
Theoretical framework.

The research model in Figure 1 follows the classic information processing framework model of “exposure → attention → interpretation → memory” (Hansen et al., 2006). Exposure means that advertising appear in the human visual nerve within the scope. Attention means that the receiving nerve transfers the perceived advertising information to the brain for processing to form an advertising attitude. Interpretation means to give meaning to the received advertising information to form a self-brand connection. Finally, memory refers to the advertising attitude connotations (such as brand promotion) are used in the short-term (such as purchase) or retained in the long-term (brand attitude).

## Methods and Results

### Participants and Procedures

In order to avoid the influence of brand awareness on the advertising effect value, this study adopted the sports brand “Li Ning” familiar to the participants, and the advertising slogan was “The old place, waiting for you.” The first reason for choosing this advertising was that the advertising was only launched a week before we conducted the questionnaire test, and the participants were able to recall the channels of receiving advertising more clearly. The second reason was that the target population of the product in China also included a large number of news feed advertising samples and traditional display advertising samples.

Because the test advertising are video advertising, this study used online network survey methods to obtain data. *We formed a questionnaire link through the questionnaire star platform. Participants use their mobile phones to scan the QR code of wechat and fill in the answer on site. The time for filling in the answer generally does not exceed 10 min. After filling in the answer, everyone will receive a red envelope of RMB10. We don’t distinguish the categories of participates. Even those participants who have not seen the advertisement and do not belong to our research object will give them RMB 10 in return.* Specifically, researchers randomly selected subjects at shopping malls or subway entrances. The researchers introduced the research content to them and told them that there was no ethical risk in this survey and they could withdraw at any time. They were asked to complete the electronic questionnaire only with their consent. The participants first needs to watch the video advertising inserted in the electronic questionnaire and fill in the media category in which the advertising has been seen recently before further answering the question. They will see two media categories, that is, news feed advertising and traditional advertising. Based on the research of Kanuri et al. (2018), we distinguish between news feed advertising and traditional advertising from the presentation of advertising. News feed advertising are those embedded in social streaming media platforms, news streaming media platforms, and video streaming media platforms (e.g., WeChat, Tiktok). The corresponding samples are called “news feed advertising samples.” Traditional display advertising are embedded in traditional media (e.g., television, newspapers, magazines). The corresponding samples are called “traditional display advertising samples.” Because the purpose of this study is to compare the differences between news feed advertising and traditional advertising, this study excludes the samples that have seen both news feed advertising and traditional advertising, and also excludes the samples that have not seen either of the two media. The participants can submit the question only after completing the questionnaire, so there is no missing value.

### Measures

The three latent variables (e.g., advertising attitude) are all measured using a 5-point scale. Participants rated their advertising attitude (α = 0.89) and brand attitude (α = 0.91) using the scale developed by Homer (2009). Specifically, a series of ratings of the experience (six five point semantic differential items anchored by pleasant/unpleasant, enjoyable/not enjoyable, dull/exciting, interesting/boring, usual/unusual, and real/fake) to measure advertising attitude, followed by a series of five point semantic differential items (such as “1 = unfavorable, 5 = favorable”) for brand attitude. Then, participants rated their self-brand connection using the scale developed by Jennifer E. Escalas and Bettman (2003) (α = 0.96), and an example item is “I feel a personal connection to Lining.” Brand experience was measured using status variables (1 = never experienced; 2 = ever experienced; 3 = always experienced).

### Sample Characteristics and Correlations

After excluding samples that have not seen the advertising and have seen the advertising through traditional display advertising and news feed advertising at the same time, a total of 408 valid samples were obtained. 139 samples were exposed to “traditional display advertising” (34.07%), and 269 samples exposed to “news feed advertising” (65.93%). The questionnaire IP showed that the samples were from 14 provinces in China (e.g., Sichuan, Liaoning, Yunnan, and Shanghai). Among the 408 samples, approximately 54.17% are male. From the perspective of age distribution structure, there are a total of 176 people (43.14%) in the sample under 30 who are more likely to be exposed to news feed advertising, and a total of 117 people (28.68%) in the sample over 45 who are more likely to access traditional advertising. It can better represent the recipients of news feed advertising and traditional advertising (see Table 1). Table 2 shows the descriptive statistics and correlations of the variables involved in the conceptual model.

**TABLE 1:** Demographic distribution characteristics of the samples.

**Age**	**Gender**		**Frequency**
	**Female**	**Male**	
Under 18 years old	3	2	5 (1.23%)
18–30	88	83	171 (41.91%)
30–35	51	64	115 (28.19%)
Over 50 years old	45	72	117 (28.68%)
Frequency	187 (45.83%)	221 (54.17%)	408 (100.00%)

**TABLE 2:** Descriptive statistics and correlations.

**Variables**	**Mean**	**Std. Dev.**	**Min**	**Max**	**1**	**2**	**3**	**4**	**5**
1. Type of Advertising	0.34	0.47	0	1	1.00				
2. Brand Experience	2.11	0.71	1	3	0.15***	1.00			
3. Advertising Attitude	3.85	0.89	1	5	0.31***	0.22***	1.00		
4. Self-brand Connection	3.31	0.92	1	5	0.24***	0.34***	0.58***	1.00	
**5. Brand Attitude**	4.00	0.79	1.5	5	0.25***	0.29***	0.69***	0.62***	1.00

**n* = 408.*

***p < 0.001, **p < 0.05; ***p < 0.01.

### Study 1

This study used the propensity score matching methods to deal with the effect of news feed advertising on the advertising attitudes and brand attitudes. This is mainly due to the following two reasons. First, traditional methods can only obtain accurate effect values when dealing with random experiments. The cost of random experiments is too high for this study. Second, propensity score matching methods can rebalance the data to make them more similar to the data generated by randomization (Guo and Fraser, 2015).

In order to obtain more information from closer matchers, this study used the Kernel Matching method. Specifically, k-Nearest Neighbor matching (*k* = 4) was used. The matching variables included the following four variables: the main medium for obtaining information (1 = mobile phone; 2 = TV; 3 = computer; 4 = others), sports product purchase experience (1 = never purchased; 2 = occasionally purchased; 3 = often purchased), gender and age.

**FIGURE 2:**
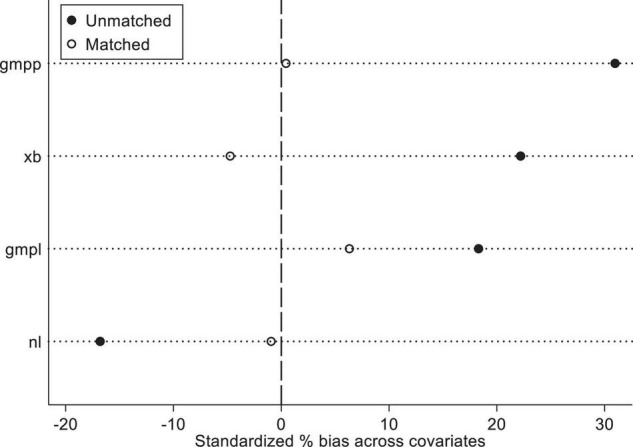
Comparison of covariate bias before and after matching.

Table 3 shows that the treat effect of unmatched samples (news feed advertising vs. traditional advertising) was significant. The influence of news feed advertising on advertising attitude and brand attitude was significantly higher than traditional advertising (advertising attitude: Δ*M*_11_ = 0.58, *t* = 6.54, *p* < 0.01; brand attitude: Δ*M*_21_ = 0.42, *t* = 5.27, *p* < 0.01). After matching analysis, the average treatment effect on the treated (ATT) of advertising attitude and brand attitude decreased, but it was still significant (advertising attitude: Δ*M*_12_ = 0.51, *t* = 6.54, *p* < 0.01; brand attitude: Δ*M*_22_ = 0.37, *t* = 5.27, *p* < 0.01). It could be seen from Table 4 that a total of 9 sample values failed to participate in the matching analysis, of which 8 samples were from the “traditional display advertising sample” group. At the same time, it could be seen from Figure 2 that the covariance quality of covariates has been significantly improved after matching.

**TABLE 3:** Analysis on the effect value of news feed advertising.

	**Brand attitude**			**Matching image perception**		
	**Difference**	**SE**	**T**	**Difference**	**SE**	**T**
**Match by default error (*n* = 408)**						
unmatched	0.58	0.09	6.54***	0.42	0.08	5.27***
ATT	0.51	0.09	5.39***	0.37	0.09	4.19***
ATU	0.52			0.34		
ATE	0.52			0.35		
**Bootstrap was used for matching (Replications = 1,000)**						
ATT	0.51	0.09	5.39***	0.37	0.09	4.18***
ATU	0.52	0.09	5.58***	0.34	0.09	3.78***
ATE	0.52	0.09	5.92***	0.35	0.08	4.22***

**p < 0.05; ***p < 0.01.

*Using the command of psmatcha2 in Stata software to analyze.*

**TABLE 4:** Propensity score variable matching.

**psmatch2: Treatment assignment**	**psmatch2: Common support**	
	**Off**	**On**
Untreated	8	261
Treated	1	138
Total	9	399

In order to obtain the standard error of ATU (Average Treatment Effect on the Untreated) and ATE (Average Treatment Effect), this study used bootstrap to analyze. In bootstrap analysis, 10101 random seeds were set, and 1000 repeated samples were calculated. The results showed that the effect values (ATT, ATU, ATE) of treat (news feed advertising vs. traditional advertising) on advertising attitude and matching image perception were statistically significant, thus, H1 and H2 (a) were supported.

In order to test whether there is hidden bias (i.e., what the unmeasured covariate would have to be like to alter the conclusion of the study), we conducted Rosehanm’s sensitive analysis on the above analysis conclusion (Guo and Fraser, 2015). The results show that the conclusion began to become sensitive (*P*_max_ = 0. 098) when Γ = 2.4 (greater than the critical value of 2). Therefore, the research conclusion was not sensitive to hidden bias.

### Study 2

Study 1 supported the hypothesis that news feed advertising has a stronger positive impact on advertising attitude and brand attitude than traditional advertising. Study 2 will establish the path analysis with observed variables (PA-OA) model to analyze the multiple correlations between variables in Figure 1.

#### Model Validation

This study used the PA-OA model with Stata15.0 to test the mediating effect of advertising perception, self-brand connection and the moderating effect of brand experience. Table 5 shows that the model fits well.

**TABLE 5:** Statistical summary of model fit.

**Model goodness of fit indices**	**Recommended value**	**Results in this study**
LR test of model vs. saturated [*x*^2^(2)]	Sig > 0.05	0.311
Root Mean Squared Error of Approximation (RMSEA)	0.05–0.08	0.020
Standardized root mean squared residual (SRMR)	<0.05	0.012
Tucker-Lewis index (TLI or NNFI)	≥0.9	0.995
Comparative Fit Index (CFI)	≥0.9	0.999

#### Hypotheses Test

**FIGURE 3:**
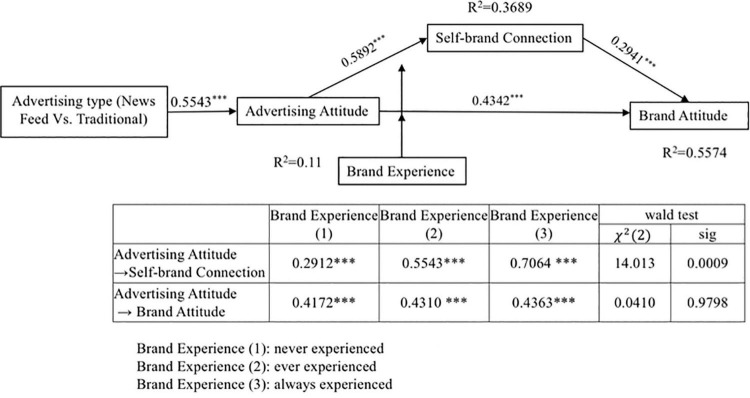
Relationships between constructs. ***p < 0.01, **p < 0.05.

As shown in Figure 3 and Table 6, the indirect effect of advertising type on brand attitude [including two paths: (1) advertising type → advertising attitude → brand attitude; (2) advertising type → advertising attitude → self-brand connection → brand attitude] was significant (indirect effect = 0.3368, *p* < 0.01). The indirect effect of advertising attitude on brand attitude (advertising attitude → self-brand connection → brand attitude) was also significant (indirect effect = 0.1732, *p* < 0.01). Thus, mediation effects were supported. What’s more, the direct effect of advertising type on brand attitude was not significant (effect value = 0.0617, *p* = 0.245), and the direct effect of advertising attitude on brand attitude was significant (effect value = 0.4342, *p* < 0.01). Therefore, the advertising attitude fully mediated the relationship between advertising type and brand attitude, and self-brand connection partially mediated the relationship between advertising attitude and brand attitude. Therefore, H3 was supported.

**TABLE 6:** Hypotheses test results.

	**Hypotheses**	**Coefficients**	**Conclusion**
H2(b)	Brand attitude mediated the relationship between advertising type and brand attitude	0.3368*** (0.05658)	supported
H3	Self-brand connection mediated the relationship between advertising attitude and brand attitude	0.1732*** (0.0243)	supported

**p < 0.05; ***p < 0.01. The standard error is in brackets.

This study used group regression to test the moderating effect of brand experience. The questionnaire was divided into three groups. Group one was “never experienced” (*n* = 84), group two was “ever experienced” (*n* = 197), and group three was “always experienced” (*n* = 127). The results showed that brand experience moderated the mediating effect of self-brand connection [*x*^2^ (2) = 14.013, *p* < 0.01], but the moderating effect of brand experience on the relationship between advertising attitude and brand attitude was not obvious [*x*^2^ (2) = 0.041, *p* = 0.98]. Therefore, H4 (b) was supported and H4 (a) was not supported (see Table 7).

**TABLE 7:** Multi-group results according to brand experience.

	**Hypotheses**	**Coefficients**	**Conclusion**
H4(a)	Brand experience moderated the relationship between advertising attitude and brand attitude	0.0410	Not supported
H4(b)	Brand experience moderated the relationship between advertising attitude and self-brand connection	14.013***	supported

**p < 0.05; ***p < 0.01.

## Discussion

Brand attitude is very important for business operation, so many studies have been carried out to explore the influencing factors of brand attitude (Hoek et al., 2020). Compared with traditional display advertising, news feed advertising can bring more positive advertising attitude and brand attitude to consumers. Although there are differences between news feed advertising and traditional advertising display in terms of communication platforms, display methods, and the fluency of consumer information processing, the classical information processing process model (exposure → attention → interpretation → memory) can still better explain why news feed advertising can bring more positive results, such as positive consumer attitudes and the mechanism of their occurrence and transmission. These results indicated that consumers’ information processing procedures for news feed advertising are consistent with traditional advertising.

### Theoretical Implications

First, exposure of news feed advertising can stimulate more positive consumer attitudes (advertising attitude and brand attitude). This study found that for those consumers who do not want to accept the advertising with obvious motivation to persuade, or do not want to be disturbed, the news feed advertising is more effective as a means of disclosure. Compared with the involuntary exposure of traditional advertising, news feed advertising is more of a voluntary and self-selecting feature (so-called permission-based marketing) that consumers encounter when they are seeking information or entertainment. Permission-based marketing is characterized by high effectiveness and low intrusiveness, and it is easier to activate consumer’s intuitive thinking mode (Martins et al., 2019), so it is easier to form a more positive advertising attitude and brand attitude. Therefore, we should “learn to use a rifle instead of using an old shotgun all the time,” and actively meet the challenges and opportunities brought by streaming media advertising.

Second, compared with traditional advertising, news feed advertising can induce more positive advertising attitudes among consumers, which is related to the fact that news feed advertising is easier to stimulate consumer’s promotion focused motives. When consumers focus on promotive motivation, their attitudes toward advertisements are more abstract (intuitive thinking mode), and they are more likely to strike a chord with the emotions expressed by advertising, especially when the emotions conveyed by advertising are positive. There are three main reasons why news feed advertising can achieve a more positive promotive motivation. First, different from traditional advertising which relies on high intensity and repeated stimulation, news feed advertising has the characteristics of low interference and strong interaction, and it is not easy to stimulate consumer’s resistance to advertising. Second, in advertising practice, when regulators have weak control over traditional display advertising, excessive false advertising and false propaganda are more likely to inspire consumers to generate prevention-focused motives on traditional advertising. Third, as a “gatekeeper role,” consumers supervise the objectivity and accuracy of news feed advertising through the way of comments and forwarding, which greatly enhances the credibility of advertising.

Third, there are two paths for converting a positive advertising attitude to a positive brand attitude (memory, purchase decision, rule, etc.). The first path is that a positive advertising attitude is directly transformed into a positive brand attitude. This path does not require consumers to interpret the advertising (directly from attention to memory), and is not affected by consumer’s brand purchase experience. In other words, when consumers are motivated by promotion, their attitude toward advertising is more abstract, and it is easier to resonate with the emotion expressed in advertising, especially when the emotion of advertising is positive. Therefore, for new products or emerging markets, news feed advertising has a stronger “drainage” effect than traditional advertising. The second path is to construct a positive brand attitude through self-brand connection (interpretation). When the positive advertising attitude is formed, the news feed advertising appeal with less interference and hidden persuasive intention can better stimulate the generation of consumers’ promotion focused motives, and then promote the emotional response of consumer’s self-concept and brand connotation to form the “self-brand connection.” This path is more stable and easier to be extracted in different scenes because it adds the consumer’s emotion to the brand. However, this path is moderated by consumer’s brand experience, that is, news feed advertising needs to be combined with good product experience to form a Relational Message to achieve the best results.

### Practical Implications

This study provides critical managerial insights to managers of online advertisers from the perspective of news feed advertising. First, our research outcome can help managers understand the role of news feed advertising. Compared with traditional display advertising, news feed advertising can positively influence consumer’s brand attitude through advertising attitude. In order to achieve this goal, enterprises can optimize the advertising investment strategy from the following aspects. On the one hand, enterprises should build a more complete user portrait, and provide targeted advertising ideas according to the personalized needs of consumers. On the other hand, enterprises should highlight the significance of original advertising, that is, to provide valuable information and make advertising integrate into the overall media environment.

Second, self-brand connection plays an important role between advertising attitude and brand attitude. The results of this study show that positive advertising attitude can enhance self-brand connection, so as to build a positive brand attitude. Thus, enterprises can enhance consumer’s self-brand connection from the following aspects. On the one hand, in the process of designing advertising content, enterprises should try to consider the characteristics of user groups, so that consumers can resonate with advertisings and enhance their self-brand connection. On the other hand, enterprises should formulate different ways of advertising expression for different consumers, so as to enhance consumer’s acceptance and recognition of advertising.

Third, this study suggests that enterprises should enhance the consumer’s brand experience. When the consumer has high brand experience, the impact of the news feed advertising on brand attitude through advertising brand and self-brand connection is very more strong. Therefore, in order to enhance consumer’s brand attitude, it is an important task to improve consumer’s brand experience while managing the news feed advertising and advertising brand. Enterprises should pay special attention to the brand experience of customers, and use various means and methods to continuously improve customers’ sensory, emotional, thinking, behavior, and related experience, so as to further enhance the brand relationship. For example, enterprises organize some brand interaction activities to encourage consumers to communicate in the activities, share their own experience in the purchase process of brand goods or services, and experience after using the goods or services, so as to enhance mutual feelings and trust.

### Limitations and Future Research

This study also has some limitations. First, this study is not a strict experimental study. Although the samples were matched through propensity score analysis, so there may still be sample bias problems. Second, this study adopts the observation experiment, but the number of advertising views of the participants is not included in the control variable in the formal test. Future studies should take viewing times as a control variable to verify the relevant results of this study. Third, the experimental advertising in this study is short video advertisement, and it is not clear whether the research conclusion is applicable to picture advertisement. Third, this study does not pre-test advertising attitudes and brand attitudes, so it is not clear whether consumers’ attitudes toward advertising and brand are derived from their real advertising experience or from the advertising effect generated in the questionnaire survey. In the further research, it is necessary to use experimental research method to measure the effect of advertising, and study the effect of different advertising types on consumer attitude.

## Data Availability Statement

The raw data supporting the conclusions of this article will be made available by the authors, without undue reservation.

## Ethics Statement

Ethical review and approval was not required for the study on human participants in accordance with the local legislation and institutional requirements. This study was carried out in accordance with the ethical guidelines of the American Psychological Association with written informed consent from all subjects. We introduced the research purpose, goals, and plans to each employee and asked their permission to participate in this research.

## Author Contributions

QD contributed to designing, analyzing, and writing the study. YY contributed to collecting research data and writing the study. YL contributed to reviewing literature and writing the study. QH contributed to collating literature and modifying sentences. All authors contributed to the article and approved the submitted version.

## Conflict of Interest

The authors declare that the research was conducted in the absence of any commercial or financial relationships that could be construed as a potential conflict of interest.

## Publisher’s Note

All claims expressed in this article are solely those of the authors and do not necessarily represent those of their affiliated organizations, or those of the publisher, the editors and the reviewers. Any product that may be evaluated in this article, or claim that may be made by its manufacturer, is not guaranteed or endorsed by the publisher.

## References

[B1] Aribarg A. Schwartz E. M. (2020). Native advertising in online news: trade-offs among clicks, brand recognition, and website trustworthiness. *J. Market. Res.* 57 20–34. 10.1177/0022243719879711

[B2] Boerman S. C. van Reijmersdal E. A. Neijens P. C. (2012). Sponsorship disclosure: effects of duration on persuasion knowledge and brand responses. *J. Commun.* 62 1047–1064. 10.1111/j.1460-2466.2012.01677.x

[B3] Boerman S. C. Van Reijmersdal E. A. Neijens P. C. (2014). Effects of sponsorship disclosure timing on the processing of sponsored content: a study on the effectiveness of European disclosure regulations. *Psychol. Market.* 31 214–224. 10.1002/mar.20688

[B4] Boerman S. C. van Reijmersdal E. A. Neijens P. C. (2015). Using eye tracking to understand the effects of brand placement disclosure types in television programs. *J. Advert.* 44 196–207. 10.1080/00913367.2014.967423

[B5] Brakus J. J. Schmitt B. H. Zarantonello L. (2009). Brand experience: what is it? how is it measured? does it affect loyalty? *J. Market.* 73 52–68. 10.1509/jmkg.73.3.52 11670861

[B6] Campbell C. Evans N. J. (2018). The role of a companion banner andsponsorship transparency in recognizing and evaluating article-style nativeadvertising. *J. Interact. Market.* 43 17–32. 10.1016/j.intmar.2018.02.002

[B7] Campbell M. C. Kirmani A. (2000). Consumers’ use of persuasion knowledge: The effects of accessibility and cognitive capacity on perceptions of an influence agent. *J. Consum. Res.* 27 69–83. 10.1086/314309

[B8] Drèze X. Hussherr F.-X. (2003). Internet advertising: is anybody watching? *J. Interact. Market.* 17 8–23. 10.1002/dir.10063

[B9] Escalas J. E. (2004). Narrative processing: building consumer connections to brands. *J. Consum. Psychol.* 14 168–180.

[B10] Escalas J. E. Bettman J. R. (2003). You are what theyeat: the influence of reference groups on consumers’ connections to brands. *J. Consum. Psychol.* 13 339–348. 10.2307/1480222

[B11] Escalas J. E. Bettman J. R. (2005). Self-construal, reference groups, and brand meaning. *J. Consum. Res.* 32 378–389. 10.1086/497549

[B12] Escalas J. E. Bettman J. R. (2013). “Exploring the effect of self-brand connections on processing brand information as self-information,” in *The Routledge Companion to Identity and Consumption*, eds Ruvio A. A. Belk R. W. (Boca Raton, FL: CRC Press), 366–374.

[B13] Ferreira C. Michaelidou N. Moraes C. Mcgrath M. (2017). Social media advertising: factors influencing consumer ad avoidance. *J. Custom. Behav.* 16 183–201. 10.1362/147539217X14909733609398

[B14] Fulgoni G. Lipsman A. (2014). Digital game changers how social media will help usher in the era of mobile and multi-platform campaign-effectiveness measurement. *J. Advert. Res.* 54:11. 10.2501/jar-54-1-011-016

[B15] Grewal D. Bart Y. Spann M. Zubcsek P. P. (2016). Mobile advertising: a framework and research agenda. *J. Interact. Market.* 34 3–14. 10.1016/j.intmar.2016.03.003

[B16] Guo S. Fraser M. W. (2015). *Propensity Score Analysis: Statistical Methods and Applications*, 2nd Edn. Thousand Oaks, CA: Sage Publications, Inc.

[B17] Hansen F. Halling J. Christensen L. B. (2006). Choosing among alternative parties to be sponsored for supporting brand strategies, based upon emotional responses. *J. Consum. Behav.* 5 504–517. 10.1002/cb.199

[B18] Hervet G. Guérard K. Tremblay S. Chtourou M. S. (2011). Is banner blindness genuine? Eye tracking internet text advertising. *Appl. Cogn. Psychol.* 25 708–716. 10.1002/acp.1742

[B19] Hoek R. W. Rozendaal E. Van Schie H. T. Van Reijmersdal E. A. Buijzen M. (2020). Testing the effectiveness of a disclosure in activating children’s advertising literacy in the context of embedded advertising in vlogs. *Front. Psychol.* 11:451. 10.3389/fpsyg.2020.00451 32256430 PMC7090090

[B20] Hoffman D. L. Novak T. P. (2018). Consumer and object eperience in the Internet of things: an assemblage theoryapproach. *J. Consum. Res.* 44 1178–1204. 10.1093/jcr/ucx105

[B21] Homer M. P. (2009). Product placements. *J. Advert.* 38 21–32. 10.2753/joa0091-3367380302

[B22] Kanuri V. K. Chen Y. Sridhar S. (2018). Scheduling content on social media: theory, evidence, and application. *J. Market.* 82 89–108. 10.1177/0022242918805411

[B23] Kirmani A. Zhu R. (2007). Vigilant against manipulation: the effect of regulatory focus on the use of persuasion knowledge. *J. Market. Res.* 44 688–701. 10.1509/jmkr.44.4.688 11670861

[B24] Lee A. Y. Labroo A. A. (2004). The effect of conceptual and perceptual fluency on brand evaluation. *J. Market. Res.* 41 151–165. 10.2139/ssrn.967768

[B25] Marian F. Peter W. (1994). The persuasion knowledge model: how people cope with persuasion attempts. *J. Consum. Res.* 21 1–31. 10.1086/209380

[B26] Martins J. Costa C. Oliveira T. Goncalves R. Branco F. (2019). How smartphone advertising influences consumers’ purchase intention. *J. Bus. Res.* 94 378–387. 10.1016/j.jbusres.2017.12.047

[B27] Sahni N. S. Nair H. S. (2020). Sponsorship disclosure and consumer deception: experimental evidence from native advertising in mobile search. *Market. Sci.* 39 5–32. 10.1287/mksc.2018.1125 19642375

[B28] Sheth J. N. Solomon M. R. (2014). Extending the extended self in a digital world. *J. Market. Theory Pract.* 22 123–132.

[B29] Wang Y. John D. R. (2019). Up, up, and away: upgrading as a response to dissimilar brand users. *J. Market. Res.* 56 142–157.

[B30] Wei M.-L. Fischer E. Main K. J. (2008). An examination of the effects of activating persuasion knowledge on consumer response to brands engaging in covert marketing. *J. Public Policy Market.* 27 34–44. 10.1509/jppm.27.1.34 11670861

[B31] Wojdynski B. W. Evans N. J. (2016). Going native: Effects of disclosure position and language on the recognition and evaluation of online native advertising. *J. Advert.* 45 157–168. 10.1080/00913367.2015.1115380

[B32] Wollschlaeger M. Sauter T. Jasperneite J. (2017). The future of industrial communication. *IEEE Indus. Electron. Mag.* 11 17–27. 10.1109/mie.2017.2649104

[B33] Zeng F. Tao R. Yang Y. Xie T. (2017). How social communications influence advertising perception and response in online communities? *Front. Psychol.* 8:1349. 10.3389/fpsyg.2017.01349 28855879 PMC5557725

